# The Role of Soy in Vegetarian Diets

**DOI:** 10.3390/nu2080855

**Published:** 2010-08-06

**Authors:** Mark Messina, Virginia Messina

**Affiliations:** 1 Department of Nutrition, School of Public Health, Loma Linda University, Loma Linda, California, USA; 2 Nutrition Matters, Inc., Port Townsend, WA 98368, USA; Email: messina@olympus.net

**Keywords:** soya, isoflavones, coronary heart disease, cancer, vegetarian, osteoporosis, safety, breast cancer, feminization, intake

## Abstract

Soyfoods have long been prized among vegetarians for both their high protein content and versatility. Soybeans differ markedly in macronutrient content from other legumes, being much higher in fat and protein, and lower in carbohydrate. In recent years however, soyfoods and specific soybean constituents, especially isoflavones, have been the subject of an impressive amount of research. Nearly 2,000 soy-related papers are published annually. This research has focused primarily on the benefits that soyfoods may provide independent of their nutrient content. There is particular interest in the role that soyfoods have in reducing risk of heart disease, osteoporosis and certain forms of cancer. However, the estrogen-like effects of isoflavones observed in animal studies have also raised concerns about potential harmful effects of soyfood consumption. This review addresses questions related to soy and chronic disease risk, provides recommendations for optimal intakes, and discusses potential contraindications. As reviewed, the evidence indicates that, with the exception of those individuals allergic to soy protein, soyfoods can play a beneficial role in the diets of vegetarians. Concerns about adverse effects are not supported by the clinical or epidemiologic literature. Based on the soy intake associated with health benefits in the epidemiologic studies and the benefits noted in clinical trials, optimal adult soy intake would appear to be between two and four servings per day.

## 1. Introduction

While soyfoods are relatively new to U.S. consumers, they have been consumed in Asia for centuries. Among western vegetarians, they have been popular for many decades, largely because of their nutrient content but also because they are extremely versatile as alternatives to meat, dairy products, snack foods, and can even count toward daily servings of vegetables. Although the quality of soy protein is higher than that of other legumes (protein digestibility corrected amino acid scores for soy protein from different soyfoods ranges from about 0.9 to 1.0) [1] and soybeans are the only legume to provide ample amounts of the essential omega-3 fatty acid, α-linolenic acid (ALA) [2], they have often been valued by vegetarians more for the ease with which they can be incorporated into the diet than their nutrient content. However, research over the past two decades has led to renewed interest in the health and nutritional aspects of soyfoods that focuses on much more than their protein and fat content. 

Approximately 2,000 soy-related papers are published annually and the focus of much of this research is on chronic disease prevention. There is particular interest in understanding the possible protective effects of soy against heart disease [3], osteoporosis [4,5], and certain forms of cancer [6,7] as well as the impact of soy on menopausal hot flushes [8]. Interest in these relationships is fueled in large part by the fact that, among commonly consumed foods, soybeans and the foods made from them are essentially unique dietary sources of isoflavones [9]. More than half of the soy-related papers published each year specifically involve these soybean constituents, which are naturally-occurring diphenolic compounds that bind to estrogen receptors [10].

Although isoflavones have been associated with certain health benefits, they are also at the heart of some of the recent controversy surrounding soyfoods and have given rise to some uncertainty among both vegetarians and health professionals about the merits of adding soy to the diet [11]. Since vegetarians and especially vegans are typically high-soy consumers, it is important to address the health consequences of adding soyfoods to the diets of those eschewing all or at least some types of animal products from their diet. 

This manuscript addresses questions related to soy and chronic disease risk, provides intake recommendations and discusses potential contraindications. Since there are now ample amounts of clinical and epidemiological data to address these issues, and the ability to extrapolate the results from rodents to humans is always a matter of debate, the human data will be the primary focus of this review. 

## 2. Understanding Isoflavones

Isoflavones are a subclass of flavonoids but with a much more limited distribution in nature. In soybeans and non-fermented soyfoods, isoflavones occur primarily as β-glycosides (less than 2% is present as the aglycone). The three soybean isoflavones, genistein, daidzein and glycitein and their various glycoside forms account for roughly 50, 40, and 10 percent respectively, of total isoflavone content. A general rule of thumb for estimating the isoflavone content of a traditional soyfood is to multiple the grams of protein in that food by 3.5 mg [12] This ratio does not hold for highly processed soy products since during processing considerable isoflavone content can be lost [13,14]. Throughout this text, isoflavone weights refer to the aglycone equivalent amounts [12].

Plasma isoflavone levels increase in direct relation to the amount ingested, although at higher intakes absorption may become somewhat less efficient [15,16]. Because their half life is approximately 6 to 8 h, within 24 h of consuming soyfoods, most isoflavones are excreted from the body [17]. Isoflavones bind to estrogen receptors, albeit more weakly than estrogen, and affect estrogen-regulated gene products, and for this reason are referred to as phytoestrogens [18]. However, because isoflavones bind with much greater affinity to and more effectively transactivate estrogen receptor (ER) beta (ERβ) in comparison to ERα they are generally classified as natural selective estrogen receptor modulators (SERMs) (mixed estrogen agonists/antagonists) [10]. SERMs, such as the breast cancer drug tamoxifen and the osteoporosis drug raloxifene, have estrogen-like effects in some tissues but either no effects or antiestrogenic effects in other tissues in which estrogen receptors are present [19]. Not surprisingly, research shows that isoflavones affect the expression of many genes differently than estrogen [19]. 

Independent of ER binding, isoflavones and especially genistein, hold the potential to exert physiological effects since they affect signal transduction pathways by inhibiting the activity of many enzymes (e.g., tyrosine protein kinase, mitogen activated kinase, and DNA topoisomerase) and regulating cellular factors that control the growth and differentiation of cells [20,21]. However, the relevance of many of these effects is unclear as the *in vitro* concentrations at which these effects occur in most instances are far higher than can be achieved *in vivo*, although there is also evidence indicating that the *in vitro* data underestimate the *in vivo* potency of isoflavones [22,23]. Finally, it would be remiss to also not mention that isoflavones are frequently classified as endocrine disruptors, chemicals that alter the function of the endocrine system and potentially cause adverse health effects  [24]. This classification is however based on results from animal studies and in contrast to this research, the human data, as discussed later, are essentially completely supportive of safety.

It is widely recognized that there is a large interindividual variation in isoflavone metabolism such that, in response to the ingestion of the same amount of isoflavones, circulating levels of the parent isoflavones and their metabolites differ markedly [25]. The conversion of the soybean isoflavone daidzein into the equol, which is accomplished by intestinal bacteria and appears to be a lifelong attribute with the exception of a temporary loss following exposure to antibiotics [26], may be a particularly important interindividual metabolic difference since equol has been proposed as being an especially beneficial compound [27]. 

Although 50% of Asians possess equol-producing bacteria only 25% of Westerners do [26]. However, a small study that included 41 and 12 Western vegetarian and nonvegetarian adults respectively, found that although as expected 25% of the latter produced equol, 59% of the former did [28]. The reason for this difference between vegetarians and nonvegetarians is unknown but obviously indicates the intestinal microbiota differs between these groups. In any event, this preliminary finding suggests that if equol-producers are more likely to benefit from soyfood consumption than non-producers, vegetarians may benefit from soy consumption more than their nonvegetarian counterparts. Those dietary factors that impact equol production have not been definitively identified [29]. 

## 3. How Much and What Kinds of Soyfoods are Consumed in Asia?

Asian soy consumption can serve as one guide for Western vegetarian soy intake recommendations. However, there is confusion among health professionals about the amount of soy consumed in Asia. Popular sources have suggested that among Asians soyfoods are used primarily only as condiments and consumed almost exclusively in fermented forms. Both of these statements are without merit. Not surprisingly however, there is a wide range of soy intake among Asian countries and even among regions within the same country. 

Within the past 15 years data from large Asian cohort, case-control and cross-sectional studies that include thousands of individuals have provided information about usual soy intake. These studies have typically administered validated food frequency questionnaires designed to comprehensively evaluate soy intake [30,31]. A 2006 review [12] that included 5 studies involving older adults in Japan [32,33,34,35,36], found soy protein intake in women ranged from a low of 6.0 g/day [35] to a high of 10.5 g/day [34], whereas the range in males was 8.0 g/day [36] to 11.3 g/day [34]. Soyfoods contributed from 6.5% [35] to 12.8%  [34] of total protein intake. Mean isoflavone intake ranged from about 30 to 50 mg/day [12,30,31]. For comparison, one serving of a traditional soyfood provides from about 7 to as much as 15 g protein per serving.

According to food disappearance data from the Food and Agricultural Organization, per capita soy protein intake has remained relatively constant during the past 40 years in Japan. However, as a percentage of total protein intake it has decreased from about 13 to 10% [12] because of the increased protein content (mostly from animal sources) of the Japanese diet. Since soy intake is decreasing among younger Japanese, absolute per capita intake may slowly begin to decline. In comparison to Japan, soy intake of Hong Kong Chinese is about only half as much [37]. Korean intake appears to be between that of Japan and Hong Kong [38]. 

In mainland China, estimating mean soy intake is more difficult since the population is heterogeneous with regard to dietary behavior. There are, however, excellent data for Shanghai, where soy intake appears to be higher than in other parts of the country. The Shanghai Men’s Health Study (SMHS) and the Shanghai Women’s Health Study (SWHS) are prospective epidemiologic studies, each involving approximately 50,000 subjects [39,40,41]. These studies indicate that daily mean soy protein and isoflavone intakes are similar to [39,40] or somewhat higher than in Japan [41]. For example, in the SMHS, for protein and isoflavones, the mean ± SEM were 12.5 and 7.94 g/day, respectively and 36.2 and 24.4, respectively [41]. There are also excellent data on the upper range of soy intake in Shanghai. In a report from the SWHS, about 10% of women consumed daily about 20 g soy protein and 85 mg isoflavones, whereas about 2% consumed ≥25 g/day soy protein (mean isoflavone intake in this group was 145 mg/day) [39]. In another report from Shanghai, among those women consuming a more plant- rather than meat-based diet, fourth quartile soy protein intake was 17 g/day [42]. Finally, in a study involving almost 3,000 Shanghai men, the overall and fourth quartile soy protein intake means were 7.8 and 16.3 g/day, respectively [43]. 

These upper intake data are consistent with that reported in some but not all Japanese studies. For example, in a case control study involving 1400 participants, Ahkter *et al.* [31] found that the mean fourth quartile (n = 174) isoflavone intake was 78.5 mg/day (estimated soy protein intake, ~20 g). In Japan, tofu, miso, natto, and fried tofu, account for about 90% of total soy protein and isoflavone intake [44,45] whereas in Shanghai, soymilk, tofu, and processed soy products other than tofu accounted for about 80% of total soy protein intake [46]. 

## 4. Vegetarian Soy Intake

Per capita US soy protein intake was estimated in one survey that included >100 foods to be only 2.2 g/day and most of the intake came from the consumption of foods to which very small amounts of soy protein had been added to traditional Western foods for functional (to improve shelf life and organoleptic properties), not nutritional reasons [47]. Not surprisingly, vegetarian soy intake is higher than that of nonvegetarians although there are relatively little published data and the findings from those studies that are available suggest that vegetarian intake, especially by lactoovovegetarians, who consume both dairy products and eggs, is lower than commonly perceived. 

In a study of soy users participating in the Adventist Health Study-2, mean (±SD) soy protein (g/day) intake among nonblack subjects as assessed by 24 h recalls (n = 33) and food frequency questionnaires (n = 42) was 12.12 ± 10.80 and 9.43 ± 7.83, respectively, although it is not clear if all of the soy users were actually vegetarians [48]. Much lower estimates of intake come from a small study that included 26 Canadian premenopausal vegetarian (no definition of vegetarian provided) women; mean (±SD) isoflavone intake (mg/day) was only 13.7 ± 18.9, which likely represents a soy protein intake of approximately 5 g/day [12]. Even still lower estimates of mean isoflavone intake–7.4 (n = 9) mg/day in one [49] and 10.5 (n = 35) in another [50], were reported by two small British studies. 

Valuable insight into vegetarian soy intake and especially differences between vegan and nonvegan vegetarians comes from another British study that was much larger in size as it included 361 nonvegetarian, 570 vegetarian, and 102 vegan pre- and post-menopausal women selected from the Oxford arm of the European Prospective Investigation into Cancer and Nutrition (EPIC) study [51]. Among this cohort, 14 and 73% of the vegetarians and vegans consumed an average of 11.2 g/day soy protein, respectively. Among the vegans, only 27% consumed fewer than 5.9 g/day whereas this was true for 86% of the vegetarians [51]. Another European study, but from Germany, found that mean (±SD) total soy intake (g/day) among strict (n = 98) and moderate vegans (n = 56) was 102 ± 99.1 and 56.8 ± 85.4, respectively [52]. It is difficult to estimate the amount of soy protein consumed from these figures without knowing specifically which soyfoods were consumed but a reasonable estimate would be about 10 and 5 g/day, respectively [12]. Finally, two US studies that included only 15 (lactoovovegetarians and vegans) and 22 vegetarians reported that mean daily isoflavone intakes were approximately 20 [53] and 15 mg, respectively [54], although the later study was conducted prior to the peak rise in soyfood consumption. 

In conclusion, a reasonable estimate is that Western vegans typically consume about 10 to 12 g/day soy protein, which is similar to the intakes of nonvegetarian Japanese and Shanghai Chinese whereas nonvegan vegetarians consume about half of this amount [12].

## 5. Role of Soy in Reducing Vegetarian Risk of Chronic Disease

### 5.1. Cardiovascular Disease

Traditional soyfoods are high in polyunsaturated fat [2] and so when substituted for foods high in saturated fat they can reduce blood cholesterol levels and risk of coronary heart disease (CHD) [55,56]. In addition to the fatty acid advantage, soy protein directly lowers blood cholesterol levels, a property formerly recognized in the form of a health claim issued by the US Food and Drug Administration (FDA) in 1999 [57]. Similar claims have been approved in several other countries including the United Kingdom [58]. The hypocholesterolemic effects of soy protein were first brought to the attention of the medical community with the publication of a meta-analysis in 1995 which found that soy protein lowered low density lipoprotein cholesterol (LDLC) by 12.9 percent [59]. 

However, within the past 5 years the results of several analyses of the literature have concluded that the cholesterol-lowering effects of soy protein are much more modest, with most studies showing LDLC reductions of 3 [60] to 5 [61] percent. The FDA is currently reevaluating the evidence in support of the health claim although they have stated that this reexamination was undertaken because of the large number of clinical trials published since the claim was first approved, not because the data are no longer supportive. 

The FDA set 25 g/day as the threshold soy protein intake required for cholesterol reduction. This suggests that soy protein is not a factor contributing to the relatively low vegetarian plasma cholesterol levels, since as discussed, few vegetarians consume this much soy [62]. Nevertheless, there is both clinical and epidemiologic evidence suggesting that it may in fact be [63,64]. Note that the FDA established 25 g/day as the threshold intake not because lower amounts were not efficacious but because few intervention studies used less than this amount. 

In the previously cited study that included 361 nonvegetarian, 570 vegetarian, and 102 vegan pre- and postmenopausal women selected from the Oxford arm of the EPIC, soy protein intake was shown to be inversely related to plasma total and LDLC levels [51]. This association remained after adjusting for a range of potentially confounding factors including saturated and polyunsaturated fat intake. Compared with women who consumed <0.5 g/day soy protein, those who consumed ≥6.0 g/day (mean, 11.2 g/day) had a 7.5% lower mean total cholesterol concentration, a 12.4% lower mean LDLC concentration, and a 9.0% lower mean ratio of total to high density lipoprotein cholesterol (each *P < *0.01). The inverse association between soy protein and cholesterol was somewhat stronger in postmenopausal than premenopausal women and in nonvegetarian compared to vegetarian and vegan women, although there were no significant interactions between diet group and soy-protein intake in relation to plasma lipid concentrations. 

Several Asian epidemiologic studies have also found inverse relationships between soy protein and blood cholesterol levels at intakes far less than 25 g/day [36,65]. In addition, a few clinical studies have reported cholesterol reductions in response to as few as 15 g/day soy protein, although as noted previously, most studies have used at least 25 g [58]. It is certainly possible that despite controlling for confounding variables the epidemiologic studies are actually identifying a healthy-user effect, *i.e.*, soy consumers eat more overall cholesterol-lowering diets than non-users. Alternatively, it may be that the inverse associations noted in the epidemiologic studies reflect lifelong soy intake (versus the relatively short term intervention trials) and/or that the hypocholesterolemic effects of the types of soyfoods consumed by participants are greater than that of isolated soy protein (by definition, ISP ≥ 90% protein), the intervention product generally used in the clinical studies. However, there is no evidence in support of any of these proposed explanations. In fact, the only study that directly compared soymilk made using ISP with soymilk made from whole soybeans found no differences in hypocholesterolemic effects [66].

Incorporating soyfoods into diets that include multiple cholesterol-lowering dietary factors provides impressive benefits as seen with the portfolio diet championed by Jenkins and colleagues [67,68,69]. Their dietary approach, in which soyfoods provide most of the protein, has been shown to result in decreases in LDLC ranging from 20 to 30 percent. In addition to providing high-quality and hypocholesterolemic protein, full-fat soyfoods have a favorable fatty acid profile and are sources of both essential fatty acids. The fat content of soybeans is comprised of approximately 44–62 linoleic acid, 21–40 oleic acid, 9–22 saturated fat, and 1–9% ALA [70,71]. 

Although the short chain omega-3 fatty acid ALA may not provide all of the proposed coronary benefits of the longer chain omega-3s, eicosapentaenoic (EPA) and docosahexaenoic (DHA), such as triglyceride lowering [72], it does appear that ALA has independent coronary benefits [73] even though the conversion of ALA to EPA is relatively poor and the conversion of EPA to DHA even poorer [74,75]. For example, a recently published cross-sectional study that included 353 male twins found that a one gram increment in habitual ALA intake was associated with an 11% decrease in plasma soluble interleukin-6 receptor concentrations, which by virtue of decreasing inflammation, could reduce CHD risk [76]. The reader is referred to the references for perspectives on whether vegetarians are at a disadvantage by not consuming DHA and EPA-containing fish  [77,78]. To some extent this question has become less relevant since vegan sources of DHA [79] and EPA are available and a new soybean oil will soon be commercially available that is 4x higher in the omega-3 fatty acid stearidonic acid, the conversion of which to EPA is about 4 times more efficient than the conversion of ALA to EPA [80]. 

Finally, there is an intriguing body of epidemiologic evidence linking soy intake with marked reductions in the risk of cardiovascular disease events, beyond that which could be attributed to the coronary benefits of ALA and cholesterol reduction [46,81,82,83]. Clinical studies have shown that soyfoods and/or isoflavones favorably affect several CHD risk factors although, with one exception, the evidence is either too limited or inconsistent to draw conclusions about potential benefits [3]. The exception is the improvement in endothelial function in postmenopausal women [84]. A recent meta-analysis found that the inconsistent results from studies evaluating this parameter is due the differing baseline health status of the subjects; isoflavones are consistently efficacious in subjects with impaired but not normal endothelial function at baseline [84]. Thus, even for vegetarians whose cholesterol levels are low, incorporating soyfoods into the diet could further reduce CHD risk.

### 5.2. Cancer

The possible chemopreventive effects of isoflavones have been rigorously investigated for 20 years since the US National Cancer Institute first funded research in this area [85]. Initially, most cancer-related research focused on breast cancer, in part because of the historically low breast cancer incidence rates in soyfood-consuming countries although in more recent years, there has been much investigation into the role of soy in reducing risk of prostate cancer, which also occurs at lower rates in Asian countries [86]. The relevant epidemiologic data have been analyzed extensively although doing so has been unnecessarily complicated by the inclusion of Western studies. Because isoflavone intake among typical Western cohorts is generally less than 1 mg/day any associations identified in these studies are unlikely to have a causal basis [87]. Therefore, these studies should be excluded from consideration.

One exception to this generalization is the EPIC-Norfolk cohort (n = 37,643) study which oversampled for vegetarians and as such, 31% of the women were classified as vegetarians, although mostly nonvegans [88]. In one report which focused on breast cancer, during the 7.4 year follow-up period 585 cases were identified. Total median isoflavone intake among the vegetarians and nonvegetarians was 10.1 and 0.23 mg/day, respectively. With the lowest (<10 mg/day; median intake, 0.2 mg/day) isoflavone intake group as the reference, the multivariable-adjusted hazard ratios for those with a moderate (10– <20 mg/day; median intake, 10.8 mg/day) or high (20+ mg/day; median intake, 31.6 mg/day) intake of isoflavones were 1.08 [95% confidence interval (CI), 0.85–1.38] and 1.17 (0.79–1.71), respectively. Thus, isoflavone intake was unrelated to breast cancer risk. In contrast, a recent meta-analysis by Wu *et al.* [6] that included one cohort study and seven case-control studies found a significant trend of decreasing risk with increasing soyfood intake even though the isoflavone tertile cutoffs were similar to those in the EPIC cohort. Compared to the lowest level of soyfood intake (median isoflavone intake, ~5 mg), the odds ratio (OR) was intermediate [OR, 0.88; 95% CI, 0.78 to 0.98] among those with modest intake (median isoflavone intake, ~10 mg isoflavones/d) and lowest (OR, 0.71; 95% CI, 0.60 to 0.85) among those with high intake (median isoflavone intake, ~20 mg isoflavones/d). 

There are a number of possible explanations for the differing conclusions of the EPIC-Norfolk cohort [88] and the Wu *et al.* [6] meta-analysis, such as differences in sources of isoflavones, differences in biological response to isoflavones among ethnic groups [89], or interactions with background diet [90]. But one of the more intriguing is that the protective effect observed in Asian studies results from lifelong exposure or exposure to isoflavones early in life. In the EPIC-Norfolk study, significant soy intake, and therefore isoflavone exposure, is more likely to have begun only during adulthood, since the adoption of a vegetarian diet often occurs later in life. 

It is beyond the scope of this paper to explore the hypothesis that early soy intake reduces breast cancer risk in detail, but it has considerable support (see references for recent reviews) [91,92]. Briefly, all four epidemiologic studies that have examined this hypothesis found protective effects associated with modest soy intake during childhood and/or adolescence with reductions in risk ranging from 28 to 60 percent [93,94,95,96]. In addition, young rodents exposed to genistein even briefly develop far fewer chemically-induced mammary tumors than control animals [97,98]. The early soy intake hypothesis is consistent with the lack of favorable effects of adult soy exposure on markers of breast cancer risk (see reference for review) [99]. Overall, although still speculative, the evidence suggests that soy can reduce breast cancer risk but to derive protection, consumption must occur early in life.

There are mixed data regarding vegetarian diets and prostate cancer risk. In a cohort that included 34,192 non-Hispanic white California Seventh-day Adventist men, risk of prostate cancer was 54% greater (95% CI, 1.05 to 2.26) among nonvegetarians compared with vegetarians (mostly nonvegans) [100]. However, in EPIC-Oxford, which included 8,451, 1,631 and 5,489 British meat eaters, fish eaters and vegetarians, respectively, relative risk (RR) was reduced among fish eaters (RR, 0.57; 95% CI, 0.33 to 0.99) but not vegetarians (RR, 0.87; 95% CI, 0.64–1.18) [101]. Similarly, in a combined analysis of 5 cohort studies that included 19,406 nonvegetarian and 5,202 vegetarian men, no relationship between vegetarian eating pattern and prostate cancer mortality was noted [102]. One obvious limitation to the epidemiologic data is the lack of information about the relationship between veganism and prostate cancer risk. This literature void is potentially important for several reasons, but especially because there is intriguing but still quite speculative evidence that dairy protein and/or calcium intake increases prostate cancer risk [103,104,105,106] possibly via an increase in circulating insulin-like growth factor I levels [107]. Vegans have been shown to have lower circulating IGF-I levels than nonvegan vegetarians and non-vegetarians [108,109]. The lack of vegan data is also unfortunate since it is this group of vegetarians that likely consume sufficient soyfoods to derive the proposed protection against prostate cancer briefly discussed below. 

In 2009, Yan and Spitznagel [7] systematically reviewed 15 epidemiologic studies on soy consumption and 9 on isoflavones in association with prostate cancer risk. Soy consumption in the Asian studies in this analysis is similar to that of vegan men. The soy intake data yielded a combined RR/OR of 0.74 (P = 0.01). When separately analyzed, studies on nonfermented and fermented soyfoods yielded a combined RR/OR of 0.70 (P = 0.01) and 1.02 (P = 0.92), respectively. The isoflavone studies yielded a combined RR/OR of 0.88 (P = 0.09); however, when analyzed separately, the combined RR/OR for studies involving Asian and Western populations were 0.52 (P = 0.01) and 0.99 (P = 0.91), respectively. Research in animals supports these findings [110,111] as does a second analysis of the epidemiologic data also published in 2009 [112]. 

In addition to possibly helping prevent the development of prostate cancer there is speculative but intriguing animal and human evidence suggesting that soy may also be useful for stopping its spread. For example, a recent study reported that levels of an enzyme that allow cells to invade tissues was markedly reduced in prostate cancer patients given high-dose genistein [113]. In agreement, adding isoflavones to the diet of mice inhibited prostate tumor metastasis to the lung—the primary site of metastasis in this animal model—by 96% [114]. As noted previously, independent of estrogen receptor binding, isoflavones and especially genistein, may affect signal transduction pathways through multiple pathways thereby potentially also affecting metastasis [20,21]

Finally, several investigators have examined the effects of soy or isoflavones on levels of prostate specific antigen (PSA). PSA is used as a tool for evaluating risk and in men with prostate tumors and serum PSA concentration is proportional to prostate tumor volume [115]. No effects of soy or isoflavones were noted in healthy subjects with low PSA levels; however, in prostate cancer patients, isoflavones slowed the rise in PSA levels [116,117,118] although only one study showed an absolute decrease and in this case, isoflavones were combined with cucurmin [119]. These data, along with the preliminary evidence about inhibiting metastasis, hold open the possibility that soyfoods may be useful in the treatment of prostate cancer. 

### 5.3. Osteoporosis

The effect of the amount and type of protein on fracture risk has, for several decades, been the subject of rigorous investigation and debate [120,121]. The inverse relationship among countries between protein intake and hip fracture incidence is frequently cited in support of a critical role for protein in bone health. For example, in an analysis published in 1992 that included 16 countries, Abelow *et al.* [122] found that animal protein intake was highly positively correlated with hip fracture incidence for women over the age of 50 whereas the intake of calcium was unrelated. And in an analysis that included 33 countries, Frassetto *et al.* [123] observed a similar relationship. In addition, this analysis reported that plant protein intake was inversely related to hip fracture risk. However, there are many potential confounders that greatly weaken the utility of these types of ecological observations. Furthermore, in contrast to the findings by Abelow *et al.* [122] and Frassetto *et al.* [123], a recent systematic review of 31 cross-sectional studies by Darling *et al.* [124] found little support for a relationship between protein intake and bone mineral density (BMD) or bone mineral content (BMC). In agreement with this finding are the results of a recent prospective cohort study in which almost 1,000 elderly individuals were followed for 5 years [125]. There were positive correlations between baseline protein intake and BMC, perhaps as a result of the higher whole body lean mass of the higher-protein consumers.

There are several mechanisms by which protein can adversely affect bone health. Dietary protein directly increases glomerular filtration rate (GFR), which modestly increases calcium excretion [126]. However, claims that dietary protein adversely affects calcium balance have been based primarily on an expected increase in calcium excretion resulting from the sulfur amino acid (SAA) content of protein [127]. The SAAs methionine and cysteine are metabolized to sulfate and hydrogen, resulting in an acid ash, which explains why dietary protein intake is correlated with renal net acid excretion. Because the skeletal system is the largest source of alkali in the body, bone is dissolved in response to production of hydrogen ions, which allows phosphate to be released as a buffering agent [128]. 

In theory therefore, because the SAA content of soy protein on a mg/g basis is lower than that of animal proteins, consuming soy protein in place of animal protein should improve calcium balance [129]. And in fact, several older studies found this to be the case [130,131]. However, in recent years the adverse effects of SAA on bone haven’t been demonstrated in clinical studies evaluating calcium balance [132,133] or in epidemiologic studies evaluating BMD [124] and fracture risk [134]. Likewise, clinical studies haven’t shown that soy protein improves calcium balance in comparison to animal protein [133,135]. Nevertheless, soyfood consumption may still help vegetarians lower their risk of osteoporosis. 

Initial speculation that soyfoods might promote bone health—at least in postmenopausal women—was based on the estrogen-like effects of isoflavones and early research showing that the synthetic isoflavone, ipriflavone, exerted skeletal benefits [136]. More than 25 trials have evaluated the effects of isoflavone-rich products on BMD in postmenopausal women although many involved small numbers of subjects and were conducted for relatively short durations [137,138]. Particularly noteworthy among this body of research, is a three-year Italian study which found that osteopenic postmenopausal women in the placebo group lost ~11% spinal BMD whereas women given 54 mg/day genistein (the amount provided by approximately 4 servings of traditional soyfoods) gained ~9% at this site [139]. Similar effects were noted at the hip. However, the striking results of this study stand in stark contrast to several recently conducted trials [140,141,142], one of which was three years in duration [143]. As to why the Italian results differ from those of most other recent studies is unclear. But it is notable that the genistein used in that study was in the aglycone form whereas in all other trials, isoflavones were in glycoside form. In any event, at this point the overall results of clinical data would have to be viewed as disappointing. 

In contrast, both of the prospective epidemiologic studies that evaluated the impact of soyfood intake on fractures found that risk was reduced by approximately one-third when women in the highest soy intake quintile or quartile were compared to women in the first [4,5]. This degree of protection is similar to that noted for estrogen therapy in clinical trials [144]. In one of the prospective studies, approximately 1,800 fractures of all types occurred in the 24,000 postmenopausal Shanghai women who were followed for 4.5 years [5]. In the other, there were almost 700 hip fractures (the only site studied) among the 35,000 postmenopausal Singaporean women during the seven-year follow up period [4]. 

As to why the two epidemiologic studies show such pronounced protective effects in contrast to the clinical studies remains to be determined. In the former, isoflavone intake occurred via the consumption of traditional soyfoods whereas the clinical studies have generally used soy extracts—although there is no evidence that this difference matters with respect to skeletal effects. It may also be that the effects noted in the epidemiologic studies result from lifelong soy intake as opposed to the relatively short-term intervention periods begun in adulthood in the clinical studies, although again, there is no direct evidence supporting this suggestion. Finally, it could simply be that the epidemiologic studies are identifying a healthy user effect as was discussed in connection to heart health. Perhaps, soy-consumers lead a bone-healthy lifestyle. 

At this point, it is clear that no conclusions about the possible skeletal benefits of soyfoods can be made. Even so, soyfoods provide high quality protein [1], which may be important for bone health [124], and some soyfoods are also good sources of calcium and vitamin D [145]. Although soymilks are typically fortified with vitamin D2, evidence indicates that vitamin D2 and D3 are bioequivalent [146,147]. Thus, soyfoods can still be part of a bone-healthy diet but whether isoflavones or soy protein offer a direct skeletal benefit remains to be determined.

### 5.4. Renal Function

The impact of dietary protein on overall renal function and GFR has been recognized for more than two decades and protein restriction has been viewed as one approach to preventing further declines in renal function in renal disease patients [148,149]. Neither dietary protein type nor amount is likely to affect renal function in healthy individuals but in those who at risk of developing renal disease—such as individuals with diabetes—dietary protein may have a big influence [149]. Depending on kidney function, current recommendations for pre-dialysis patients include a reduction in protein intake to no more than 0.6 to 0.8 g/kg body weight [150,151], which is only about 50 percent of usual US adult protein intake [152]. With the rising numbers of people at risk of developing chronic kidney disease (between 1988–1994 and 1999–2004, the prevalence of chronic kidney disease increased by 30% [153]) as a result of the increasing prevalence of diabetes, data suggesting that soy protein may favorably affect renal function relative to animal proteins have attracted attention [154,155,156,157,158,159,160,161]. For general reviews on this topic the reader is referred to the references [162,163]. 

Unlike animal protein, soy protein appears not to increase postprandial GFR or renal blood flow [154,164,165,166]. Also, some research indicates that soy protein, when substituted for animal protein, decreases urinary protein levels in individuals with chronic renal disease and in patients with diabetes [167,168,169,170,171]. In addition, recent data suggest that soyfoods may also be useful for dialysis patients [172,173,174,175] and may have advantages for renal transplant patients [176]. 

Although evidence suggests soy protein favorably affects renal function relative to animal protein, how it compares to other plant proteins has yet to be determined. However, soy protein has the advantage of being a higher quality protein than other plant proteins so its incorporation into vegetarian diets is an advantage [1]. Vegetarians are at a lower risk of developing diabetes than nonvegetarians and as such are less likely to develop renal disease [177]. But for those who are at risk, substituting soy protein for other protein sources in the diet would likely provide renal benefits. The favorable effects of soy protein on circulating lipid levels may also favorably impact renal function as evidence indicates elevated cholesterol exacerbates renal disease [158,178]. 

## 6. Mineral Balance

The mineral status of vegetarians is an issue most relevant in regard to iron, zinc and calcium although the latter is primarily an issue for vegans only. There is particular interest in understanding mineral bioavailability from soyfoods and legumes in general because of the presence in these foods of inositol hexaphosphate (phytate), which binds covalent cations thereby reducing their absorption [179,180] although there is also interest in the chemopreventive properties of phytate [181,182]. The phytate content of soybeans is higher than that of other legumes [180,183]. In general, however, the pronounced effects of inhibitors and enhancers on mineral absorption noted in acute studies aren’t nearly as apparent in long-term studies, perhaps because these factors tend to balance each other out [184]. As an aside, although fermentation leads to the hydrolysis of phytate, the extent to which fermentation of soy products affects mineral absorption is unclear [185,186,187,188,189,190,191].

The long held view is that iron absorption from soyfoods is poor, although similar to or somewhat better than from other legumes and plant foods [185,186,192]. However, new research suggests iron bioavailability from soybeans has been greatly underestimated [193]. This research, the key to which is the improved methodology used to assess absorption, indicates that iron absorption from soy is excellent. The reason is that most of the iron in soy is in the form of ferritin. Although there is some debate about the bioavailability of ferritin iron [194], two clinical studies found that in women soybean ferritin iron was highly bioavailable, essentially being equal to the absorption of iron from FeSO_4_ [193,195]. Ferritin is a large protein that reversibly concentrates iron as a solid mineral thereby making the mineral more bioavailable. Soybean varieties differ markedly in their ferritin concentration which suggests iron bioavailability from different soybean varieties may also differ [196]. Future research confirming the high bioavailability of soybean ferritin iron will require a complete reevaluation of how soyfoods are viewed as sources of iron.

When considering the impact of soy consumption on vegetarian zinc balance it is important to recognize that zinc status is much more difficult to assess than is iron status, and that soyfoods are not especially good sources of this mineral [197]. On the other hand, zinc absorption is only slightly lower from soy than from animal products. For example, zinc bioavailability from an equal mixture of ISP and milk was 47%, which was not different from the zinc bioavailability from milk (51%) although zinc bioavailability from ISP (37%) alone was about 25% lower [198]. The results from this study concur with those from several others which also show zinc absorption from soy to be similar to or about 25% lower than zinc absorption from animal foods [199,200,201,202]. As is the case for iron, both the phytic acid and protein in soy inhibit zinc absorption although in the case of zinc, the effects are much more modest [202,203,204]. 

Finally, the issue of calcium absorption from soy is particularly important for vegans, especially if calcium-fortified soymilk and calcium-set tofu are relied upon to provide large amounts of dietary calcium. Interestingly, although soybeans are high in both oxalate and phytic acid—two components that strongly inhibit calcium absorption—calcium bioavailability from soy is surprisingly good  [205]. The relatively high calcium bioavailability from soy is rather remarkable given that calcium bioavailability from legumes is generally less than 20% [206] and that the oxalate to calcium ratio in soy is similar to rhubarb and about twice as high as in spinach [207]. Calcium absorption from rhubarb and spinach is only about 9% [208] and 5% [209], respectively.

In 1991, Heaney *et al.* [210] published a seminal paper on calcium bioavailability from soybeans. Using two different strains of soybeans, they found that, in healthy premenopausal women, calcium absorption from high-phytate soybeans, low-phytate soybeans, and 2% fat dairy milk, was 31.0%, 41.4%, and 37.7%, respectively. Nearly a decade later, Heaney *et al.* [211] also reported that, in healthy men, calcium absorption from intrinsically labeled calcium-fortified soymilk was about 75% that of calcium absorption from cow’s milk (0.237 *vs.* 0.306, p < 0.01). The calcium salt used in that study was tricalcium phosphate (TCP). In 2005, the TCP findings were replicated in research by Zhao *et al.*^142^who also found that calcium absorption from soymilk fortified with TCP was about 25% lower than from cow’s milk [142]. 

However, when calcium carbonate was used as the fortificant in soymilk, calcium absorption was essentially identical to that seen with cow’s milk. Calcium carbonate is the fortificant used in most soymilk sold in the United States. Furthermore, according to Zhao *et al.* [145], because of the high amounts of TCP added, the amount of calcium available to the body from both types of calcium-fortified soymilk is similar to that from cow’s milk. In general agreement with the findings from Zhao *et al.* [145], Weaver *et al.* [212] found no difference in the absorption of calcium from tofu fortified with calcium sulfate or calcium chloride, in comparison to calcium absorption from dairy milk. 

## 7. Safety Concerns

### 7.1. Breast Cancer

Concerns that soyfoods might be harmful to breast cancer patients and women at high risk of developing breast cancer are based primarily on research showing that genistein-containing products stimulate the growth of existing estrogen-sensitive tumors in ovariectomized athymic mice implanted with MCF-7 cells, a human estrogen receptor positive breast cancer cell line [213]. The dietary genistein concentration most often used in this model is 750 ppm. The equivalent dose in humans, using the conversion based on body surface area recommended by the FDA, is about 6 mg/kg body weight [214], which is 6 to 20 fold greater than human exposure [12]. However, as discussed below the more relevant issue is not dietary exposure but circulating levels.

It is noteworthy that in this model, tumor stimulation increases with the degree to which the isoflavone product has been processed despite containing similar amounts of genistein [215]. In fact, soy flour—a minimally processed soy product—does not stimulate tumor growth in the ovariectomized athymic mouse model [215]. However, in addition to the usual limitations of rodent studies and the debatable degree to which findings from these studies can be extrapolated to humans, evidence indicates the “processing effect” does not apply to humans. 

In comparison to humans, mice have a poor ability to glucuronidate phenolic compounds such as isoflavones. Consequently, mice have markedly higher circulating levels of unconjugated genistein—the biologically active form. In the liver and intestine of animals, glucuronic acid is often linked to a wide range of substances, which by virtue of making them more water soluble, allows for their subsequent elimination from the body through the urine or feces. Furthermore, processing of soyfoods leads to even higher levels of unconjugated genistein. This is not the case in humans. 

Even more importantly, neither soyfoods nor soy-derived isoflavone supplements adversely affect markers of breast cancer risk in healthy women or breast cancer patients, such as breast tissue density and breast cell proliferation (for review see reference) [99]. This lack of effect contrasts with the effects of conventional hormone therapy, which for example, increases breast cell proliferation *in vivo* fourfold [216,217]. In addition, recent epidemiologic data indicate that post-diagnosis consumption of soyfoods improves prognosis as measured by mortality and tumor recurrence; further, no interaction with tamoxifen has been observed [218,219]. Both of these observations contrast with the animal data upon which concerns are based. Thus, while it may be premature to recommend that breast cancer patients begin to consume soy specifically for the purpose of improving prognosis, the totality of the evidence no longer justifies advice prohibiting breast cancer patients from consuming soyfoods as part of an overall healthy diet [220].

### 7.2. Feminization and Infertility

Given the large populations in many soyfood-consuming countries, it is ironic that concerns have been voiced that the estrogen-like effects of isoflavones could exert feminizing effects on men and impair fertility. Studies that lend credence to these concerns include rodent research showing isoflavone exposure lowers circulating testosterone levels and sperm concentration [221,222], a small pilot epidemiologic study that found high soyfood consumers (~3 servings per week) had lower sperm counts than non-soyfood consumers [223], and a case report describing a 60 year old man who developed gynecomastia allegedly in response to the consumption of soymilk [224]. 

However, in contrast to the effects in rodents, a recently published meta-analysis of the clinical research found that there were no effects of soyfoods or isoflavone supplements on total or free testosterone levels or levels of dihydrotestosterone—the biologically active metabolite of testosterone [225]. Similarly, the clinical evidence shows that soy does not raise estrogen levels (see reference for review) [226]. In the case report described above, the individual in question did have dramatically elevated estrogen levels (5- to 10-fold above average reference values) [224]. But this increase was in response to the consumption of 3 quarts (~12 cups) of soymilk daily, an amount (assuming it is made using the whole soybean) that would be expected to provide approximately 300 mg isoflavones (the authors suggested 361 mg) [12], an intake roughly 9 times that of the typical Japanese [12]. Clearly, excessive intake of even nutritional desirable foods can produce untoward effects. Clinical studies have shown that isoflavone intakes as high as 150 mg/day-which is still 4 times higher than typical Japanese intake—are without effect on estrogen levels [226].

Finally, in contrast to the pilot epidemiologic study cited previously, none of the three clinical studies conducted found adverse effects of soy or isoflavones on sperm or semen parameters [227,228,229]. In fact, as described in a case report published in 2004, a man with low sperm count who was unable to father a child experienced an improvement in sperm concentration and motility resulting in a successful pregnancy after being given isoflavones for six months [230]. The authors suggested that isoflavones may prove to be a treatment for oligospermia and called for clinical trials to be conducted. It is notable that in the epidemiologic study in question, in addition to having several design limitations—such as no dietary information other than soy intake being obtained—much of the reduced sperm count appeared to occur because soy use was associated with an increase in ejaculate volume [223]. It seems unlikely that this association would have a causal basis.

### 7.3. Thyroid

In *in vitro* studies and in rats, isoflavones have been shown to partially inactivate thyroid peroxidase, an enzyme required for the synthesis of thyroid hormones [231]. However, not only is the rat extremely sensitive to goitrogenic insults in comparison to humans [232,233], but despite inhibiting enzyme activity, soy-containing diets allow normal thyroid function [232,234]. Furthermore, and most importantly, a comprehensive review of the clinical literature published in 2006 found that the evidence clearly indicates that isoflavone exposure has no effect on thyroid function in euthyroid individuals [235]. Studies published subsequent to this review support this conclusion [236,237,238]. In many of these studies, isoflavone exposure was at the very high end of typical Asian dietary intake and this research now includes the results from studies 3 years in duration [239].

Soyfoods may somewhat inhibit the absorption of synthetic thyroid hormone, such as Synthroid, which is taken by hypothyroid patients [240]. However, foods in general have this effect, as do fiber-enriched foods, herbs and many drugs [241]. For this reason, thyroid hormone is taken on an empty stomach and hypothyroid patients can still consume soyfoods. If there is any small effect on absorption, the medication dose can easily be adjusted accordingly.

There are however, two relevant clinical situations related to soy and thyroid function yet to be evaluated. One involves individuals with subclinical hypothyroidism, which represents about five percent of the general adult population but a higher percentage among individuals over the age of 60 [242]. Patients with this condition have normal levels of the two primary thyroid hormones, thyroxine and triiodothyronine, but elevated levels of thyroid stimulating hormone [243]. There is no direct evidence that soyfoods pose a problem for subclinical hypothyroid patients and research specifically addressing this issue is currently underway. 

The second situation involves individuals whose iodine intake is marginal or inadequate. Ethical concerns preclude clinical research evaluating the effects of soy in such individuals. In the United States, iodine intake is generally quite good [244] but this is not the case in many parts of the world [245]. Furthermore, even in the United States, some subpopulations may not be consuming sufficient iodine, such as women of reproductive age [246]. More importantly, some data suggest that vegetarians, and especially vegans, who do not use iodized salt, may be at increased risk of developing iodine deficiency [247,248,249,250,251]. It should be noted that about 85% of sodium intake comes from processed foods and that the salt used in the making of these foods is typically not fortified with iodine. Also, dairy products provide about half of the iodine intake in the United States and other Western countries [246]. Consequently, vegans need to been especially mindful of their iodine intake, especially because they are often high-soy consumers. 

## 8. Are all Soyfoods Created Equal?

The question of whether some soyfoods are nutritionally superior to others is a reasonable and frequent one. There are clear nutrient and nonnutrient content differences among the large variety of commercially available soyfoods. For example, tempeh, soynuts and edamame, retain all of the fiber and fatty acids found in the whole soybean whereas at the other end of the spectrum, ISP, which is basically a concentrated source of soy protein, lacks these components and has a much lower isoflavone (on a mg/g protein basis) concentration than traditional soyfoods. Many processed soyfoods are high in sodium although this varies considerably among products. 

On the other hand, soymilk, which lacks soy fiber (okara), is often fortified with both calcium and vitamin D. Similarly, some types of tofu are much higher in calcium than soybeans because they are made by using a calcium salt as a coagulant. Fermentation of soybeans, such as in the making of miso and natto, leads to the creation of some potentially interesting compounds not found in nonfermented foods [252,253], but there is scant evidence to suggest fermented foods are superior to nonfermented ones in any meaningful way; in fact, the opposite case can be made on the basis of limited epidemiologic data [7,254]. On the other hand, fermentation reduces oligosaccharide content, which may reduce flatulence [255,256].

All nutritionists recommend emphasizing whole foods over more refined ones and there would appear to be no reason not to apply this dietetic principle to the consumption of soyfoods, although the extent to which different soy products have been processed spans a wide range. Furthermore, soy burgers, which are typically made with soy protein concentrate, which is ~70% protein and lacks some of the components found in the whole soybean, are certainly a nutritionally desirable replacement for hamburgers. And ISP is often a convenient way to increase the protein content of the diet. 

It is notable that many soyfoods are quite high in fat, although it is predominantly polyunsaturated. In contrast to nearly all other legumes, about 40% of the calories in soybeans come from this macronutrient [257]. However, for those desiring lower-fat soy products, many options are available. Obviously, even low-fat diets can accommodate some higher-fat foods. 

Finally, although there are many biologically active components of soybeans, as is the case for all plant foods, most evidence points toward the isoflavones, and secondarily protein, as the specific components responsible for the proposed health benefits of soyfoods. Therefore, if isoflavone intake is an important criterion in the selection of soyfoods, recognizing that processing can cause considerable isoflavone loss is an important consideration. The isoflavone concentration of some processed soy proteins may be only 20% of that found in traditional soyfoods  [14]. On the other hand, these foods still provide high quality protein and despite having a lower isoflavone concentration, can still be a source of isoflavones. In essence, essentially all soyfoods can play a role in a healthy vegetarian diet and whether specific soyfoods are emphasized will depend largely upon personal preference.

## 9. Vegetarian Soy In take Recommendations

Historically, legumes have played an important role in the diets of most cultural groups, and in many Asian countries, the most commonly consumed legume is the soybean. In contrast, legumes typically provide less than 2% of overall protein intake in Western diets [257,258,259,260]. In fact, even vegetarian legume intake is less than optimal [261]. Thus, both vegetarians and nonvegetarians would benefit by including more of these fiber- and protein-rich foods in diets. [257]. 

As discussed, soybeans are higher in protein than other legumes and their protein is of superior quality. Further, because of their versatility, soyfoods are easy to incorporate into the diet. They are also unique dietary sources of isoflavones. While much of the research on the specific benefits of isoflavones remains conflicting, those intervention studies showing health benefits have generally used between 50 and 100 mg/day of isoflavones—amounts provided by approximately two to four servings of traditional soyfoods. In Asian epidemiologic studies, benefits are generally associated with the consumption of two to three servings of soyfoods per day. Thus, evidence suggests optimal adult intake ranges from about two to four servings per day. 

With the exception of allergic reactions to soy protein, which is relatively rare among adults, there is little basis for concern that excessive amounts of soy will lead to untoward effects in healthy individuals [262]. Nevertheless, because an important dietetic principle is to consume all foods in moderation, and there is a wide variety of legumes from which to choose, a reasonable upper intake recommendation for soyfoods is four servings per day.
